# Flexible insulin therapy improves metabolic control and decreases the risk of hypoglycemia in type 1 diabetic patients

**DOI:** 10.11604/pamj.2021.40.100.18097

**Published:** 2021-10-14

**Authors:** Siham Rouf, Amine Ezzerrouqi, Salma Benyakhlef, Naima Abda, Hanane Latrech

**Affiliations:** 1 Department of Endocrinology, Mohammed VI Hospital, Medical School, Mohamed the First University, Oujda, Morocco,; 2 Laboratory of Epidemiology, Clinical research and Public Health, Mohammed VI Hospital, Medical School, Mohamed the First University, Oujda, Morocco

**Keywords:** Flexible insulin therapy, control, hypoglycemic

## Abstract

**Introduction:**

flexible insulin therapy (FIT) is considered as a crucial turning point in the management of type 1 diabetes. The purpose of this study was to evaluate the impact of this optimum therapeutic approach on improving metabolic control and decreasing hypoglycemic events in patients with type 1 diabetes.

**Methods:**

thirty-seven type 1 diabetic patients were included in a five days training programme of FIT. They had an HbA1c between 7.5 and 10%. Those patients were enrolled in a flexible insulin program and we evaluate clinical and metabolic parameters (glycated haemoglobin (HbA1c), hypoglycemic events, body mass index (BMI) and the rate of blood glucose measurements) before the course of FIT and 3, 6 and 9 months after the course.

**Results:**

over a 9 months period of the study, the frequency of mild hypoglycemia decreased from 11.7 to 1.7 episodes/3 months (p = 0.005). The baseline HbA1c value improved by 1% at 3 months with an increase of 0.2% at 6 months, which remained unchanged at 9 months (p = <0.0001). Patients who were poorly controlled (HbA1c ≥ 8%) improved their baseline HbA1c value from 9.2% to 8.0% (p = <0.0001).

**Conclusion:**

the present study confirms that a structured training programme for FIT improves glycemic control and decreases hypoglycemic events in patients with type 1 diabetes and it can be adopted in countries with weak or intermediate income (e.g. Morocco), which allows those patients to take advantages of this therapeutic approach.

## Introduction

Diabetes mellitus is a metabolic disorder characterized by an increased blood glucose level. The prevalence of diabetes in adults aged 20 - 79 years was estimated to be 8.8% in 2015 and supposed to rise to 10.4% in 2040 [1]. In terms of the International Diabetes Federation (IDF) regions, Middle East and North Africa are projected to experience the highest growth rate in the number of people with diabetes (103.8% increase in 2040) [1]. The countries with a weak or intermediate income reported an increase in prevalence of diabetes (e.g. Morocco: 1.4%) [2].

In Morocco, little data is available, considering that it belongs to the countries with no evidence of the rate estimation for type 1 diabetes. For Over 30 years, Mühlhauser and co-workers have developed the Düsseldorf model focused on teaching diabetic patients how to match their short acting insulin dose to their carbohydrate load for a better glycemic control, along with a lower risk of severe hypoglycemia [3-8]. The Diabetes Control and Complications Trial (DCCT) demonstrated that strict metabolic control in type 1 diabetes patients reduced the prevalence of microvascular complications [9]. Nevertheless, intensified insulin therapy was associated with an increase in severe hypoglycemic events [10]. Whereas, several studies showed that flexible intensive insulin therapy improved glycemic control and decreased the risk of severe hypoglycemia [11,12].

Flexible insulin therapy gives the opportunity to patients with type 1 diabetes to use carbohydrate counting to adapt their insulin doses in order to refine their dietary freedom and their diabetes self - care skills. In our Department of Diabetology at Oujda University Hospital, Morocco, we developed a 5 days educational FIT programme and it was adapted to the wide Moroccan culinary traditions especially those of the eastern region of Morocco, where our department is located. In this report, we evaluate biomedical effects of this programme including HbA1c, hypoglycemic events, BMI and the frequency of blood glucose tests on type 1 diabetic population.

## Methods

**Setting and population:** this prospective study was carried out in the Department of Diabetology at Oujda University Hospital where we developed a diabetes management program for patients with type 1 diabetes admitted in our unit for glycemic imbalance. This program starts with a basic education which includes the seven self-care behaviors: healthy eating, being active, monitoring, taking medication, problem solving, healthy coping and reducing risks, followed up by the educational program of flexible insulin therapy to allow them a greater management of their diabetes. Among one hundred and forty type 1 diabetic patients followed up in our unit, thirty-seven type 1 diabetic patients were enrolled in the educational programme of FIT. Participants maintained an inadequate glycemic control (HbA1c: 7.5 - 10%) despite the fact that they have received the basic education. The exclusion criteria were the inability to do mathematical calculations, difficulty to ensure a blood glucose monitoring, lack of motivation, psychiatric illness and pregnancy. All the enrolled patients were on basal-bolus regimens with short and long-acting insulin analogs. In our country, considered as an intermediate income country, the insulin analogs and the blood glucose checks are provided by patients, purchased at pharmacies and 100% reimbursed by the various national insurances considering that diabetes as a chronic illness requiring long-term treatment.

**Procedures:** the duration of the study was nine months; three medical visits were scheduled at 3 months interval to collect biomedical data. Patients were motivated to achieve a better metabolic control, and adjust the insulin dose depending on exercise and meals with an appropriate treatment of hyperglycemia and hypoglycemia. The FIT educational programme was taught to homogeneous group of patients according to the age and the level of understanding, over five days, from Monday to Friday. The therapeutic-education team consists of medical doctors, one dietician and a diabetes nurse educator. During this programme, the 24-hours carbohydrate fasting test permeated to adjust the long acting insulin and determine the individual effect of 10 g of oral glucose to correct a hypoglycemia. The estimation of the insulin to carbohydrate ratio was determined from insulin requirements of fast acting insulin used in the conventional regimen (the insulin units needed for 10g of carbohydrates).

In addition to the insulin - carbohydrate ratio used to cover the carbohydrates consumed at different meals, patients need to correct pre meal hyperglycemia by using a correction dose determined by the “insulin sensitivity factor” which is a dose of short acting insulin individualized according to insulin sensitivity of each patient (the effect of 1 unit of fast acting insulin on blood glucose lowering) determined by using the commonly method of “1800 rule” which is 1800/total daily dose (TDD) of long and short acting insulin = glucose rate in mg/dL decreased by administering 1 unit of short acting insulin.

**Figure 1:**
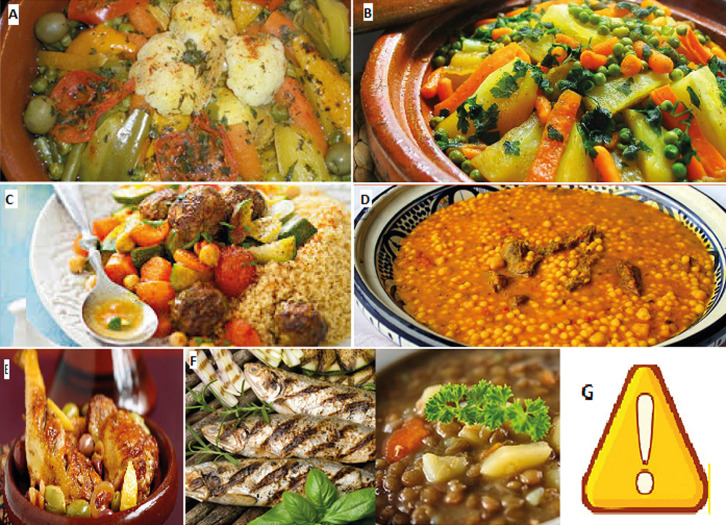
the nutritional booklet designed by our team and adapted to the Moroccan culinary traditions specifically those of the eastern region of Morocco: A) lamb or beef Moroccan tagine with vegetables (100g beef or lamb+100g vegetables): 4g carbohydrates; B) chicken Moroccan tagine with vegetables including potatoes (100g chicken+ 50g vegetables+100g potatoes): 20g carbohydrates; C) 250g Moroccan couscous (medium bowl)+ 200g vegetables: 55g carbohydrates; D) 250g berkoukech soup (the name berkoukech refers to the name of the giant couscous grains used in the soup in the berber language): 50g carbohydrates; E) chicken Moroccan tagine with olives: 10 olives: 4g carbohydrates; F) Moroccan lentil soup + fish: 250g lentils (medium bowl): 40g carbohydrates; G) pay attention to fat content of your meal

Using the insulin to carbohydrate ratio and the insulin sensitivity factor, these enable patients to adjust their pre meal glucose level to their individualized blood glucose targets. During the program, all patients were educated to use carbohydrate counting and received nutritional planning recommendations, based on providing a nutritional booklet, in Arabic language, designed by our team and adapted to the Moroccan food traditions specifically those of the eastern region of Morocco (Figure 1). This booklet illustrated carbohydrates and lipids contained in common meals and those of the Moroccan culinary traditions (e.g. Moroccan tagine). During the fifth day of the course, patients were taught to adapt their fast acting insulin to the intensity of physical activity in real life by doing a lap around the hospital accompanied by a medical doctor and a nurse. This FIT programme is evaluated five days after the course by planning a collective meal for the group of patients.

In this study, at baseline, three, six and nine months after the course of FIT, data collected included glycated haemoglobin (HbA1c), weight and BMI, blood glucose measurements and frequency of mild hypoglycemia defined as blood glucose ≤ 70mg/dl treated with fast-acting carbohydrate and severe hypoglycemia correlated with severe cognitive impairment requiring external assistance for recovery [13].

**Ethical aspects:** all patients give their informed consent to be included in the study. The data was collected anonymously and the analysis was performed with the strictest confidentiality.

**Statistical analysis:** the data as performed using Statistical Package for Social Sciences (SPSS), V21.0, and the results expressed as means ± SD. The outcomes were compared between the different follow-up time points using a mixed linear model. The Bonferroni was used to correct the p-values of the parallel comparisons of the outcomes: glycated haemoglobin, hypoglycemia, BMI and blood glucose measurements. A value of p<0.05 was considered statistically significant.

## Results

**At baseline:** participant´s average age was 20.9 years (SD 3.7, range 18-35). Twenty-five (67.6%) patients were female (Table 1). The mean duration of diabetes was 5.7 years (SD 3.7), the mean of initial BMI´s patients was 22.1 kg/m^2^(SD 2.8) and the average of HbA1c was 8.4% (SD 0.9). The daily dose of long acting insulin was decreased in 20 patients (54.4%) during the 24 hours carbohydrate fasting test. Before the course, thirty-one participants (83.8%) had the experience of mild hypoglycemia and seven patients (18.9%) had suffered at least of two severe hypoglycemia in the year preceding the course. The presence of mild hypoglycemia was not correlated with age (p = 0.15), diabetes duration (p = 0.52) or HbA1c (p = 0.71).

**Table 1:** baseline characteristics of patients

Characteristics	
Age (years), mean (SD)	20.9 (3.7)
Female n (%)	25 (67.6)
Duration of diabetes (years), mean (SD)	5.7 (3.7)
Baseline HbA1c (%), mean (SD)	8.4 (0.9)
Weight (kg), mean (SD)	61.2 (8.1)
BMI (kg/m^2^), mean (SD)	22.1 (2.8)
Total cholesterol (g/l), mean (SD)	1.5 (0.3)
Triglyceride (g/l), mean (SD)	0.8 (0.3)
HDL cholesterol (g/l), mean (SD)	0.47 (0.12)
LDL cholesterol (g/l), mean (SD)	0.8 (0.2)

HDL: high-density lipoprotein; LDL: low-density lipoprotein

**Figure 2:**
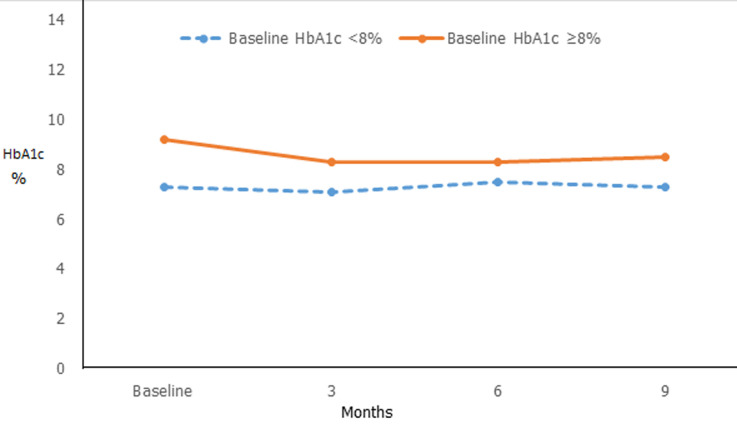
evolution of HbA1c in moderately controlled (HbA1c < 8% at baseline) and poorly controlled (HbA1c ≥ 8% at baseline) patients over the 9 months period of flexible insulin therapy program

**During the follow up:** over the 9 months period of the study, the average of HbA1c decreased significantly of 1% at 3 months moving from 8.4% (SD 0.9) to 7.4% (SD 1.1) (p = <0.0001). Nevertheless, we observed an increase of 0.2% in HbA1c at 6 months which remained unchanged at 9 months with no statistically significance difference compared to HbA1c at 3 months (p = 1.0) (Table 2). Subjects with a baseline HbA1c value over 8% achieved a greater reduction in HbA1c by 1.3% (9.2% to 7.9%) at 3 months (p = <0.0001), which remained unchanged at 6 and 9 months (Figure 2).

**Table 2:** the outcomes in the entire group at 3, 6 and 9 months

Outcomes	Baseline	3 months	6 months	9 months	p value
Hba1c (%), mean (SD)	8.4 (0.9)	7.4 (1.1)	7.6 (0.8)	7.6 (0.7)	<0.0001
BMI (kg/m^2^), mean (SD)	22.1 (2.7)	22.3 (2.9)	22.0 (2.6)	22.3 (2.7)	0.02
Mild hypoglycemia (episodes/3months), mean (SD)	11.7 (2.8)	5.6 (1.9)	2.5 (1.4)	1.7 (1.1)	0.005
Blood glucose (measurements per day), mean (SD)	2.0 (0.8)	4.5 (1.6)	4.0 (1.6)	4.9 (1.3)	<0.0001

Nonetheless, severe hypoglycemia has not been reported in the entire group, during the follow-up and the frequency of mild hypoglycemia decreased seven-fold from 11.7 (SD 2.8) episodes/3 months to 1.7 (SD 1.1) episodes/3 months which was statistically significant after 9 months (p = 0.005). Participants with a baseline HbA1c value over 8% had a significantly improvement of the rate of mild hypoglycemia (p = 0.001) (Table 3). We did not find any difference in the average of insulin to carbohydrate ratio. However, the frequency of blood glucose measurements increased three-fold from 2 (SD 0.8) times per day to 4.9 (SD 1.3) times per day (p = <0.0001).

**Table 3:** the outcomes in the poorly controlled patients (HbA1c ≥ 8%) at 3, 6 and 9 months

Outcomes	Baseline	3 months	6 months	9 months	P value
Hba1c (%), mean (SD)	9.2 (0.7)	7.9 (1.2)	7.9 (0.5)	8.0 (0.6)	<0.0001
BMI (kg/m^2^), mean (SD)	22.4 (0.8)	22.8 (0.9)	22.3 (0.8)	22.6 (0.8)	NS
Mild hypoglycemia (episodes/3months), mean (SD)	10.8 (2.6)	4.5 (1.3)	0.9 (0.4)	0.5 (0.3)	0.001
Blood glucose (measurements per day), mean (SD)	2.3 (0.2)	4.3 (0.4)	4.0 (0.3)	4.9 (0.3)	<0.0001

NS: not significant

In our study population, we observed an increase in BMI in the entire group by 0.02 kg/m^2^(p = 0.02). The lipid profile of all the included patients was normal at the baseline with no statistically difference over the 9 months period of the study (Table 1). Two patients in the entire study experienced one episode of diabetic ketoacidosis (DKA) in the year preceding the program; there was no episode of DKA in the entire group after the program.

## Discussion

The present study confirms that structured training programme for flexible insulin therapy improves glycemic control and decreases hypoglycemic events. Some type 1 diabetic patients prefer dietary restrictions, routine meals timing and consequently a lesser number of insulin injections. However, it has been reported that strict glycemic control provided by intensive insulin therapy reduced consequently the occurrence of microvascular disease [9].

The FIT educational programme takes a crucial place in metabolic control among poorly controlled patients. In our study population, the baseline HbA1c value of 8.4% showed an improvement of 1% after 3 months of the course with a slight unchanged increase of 0.2% at 6 and 9 months. Furthermore, the subgroup of diabetic patients who were poorly controlled (HbA1c ≥ 8%) decreased significantly their basic HbA1c value from 9.2% to 8.0%. Consequently, sessions were organized after the programme to remind patients of the basics of the FIT concept, especially how to determine the dose of short acting insulin by using the carbohydrate to insulin ratio and the insulin sensitivity factor according to pre-meal glucose value. In analogy with our results, a prospective study found that FIT course has positive effects on reducing hypoglycemic events despite the fact that the mean of HbA1c showed an unsustainable improvement [14].

The raising of the risk of hypoglycemia was reported as a negative effect of intensive insulin therapy in the DCCT trial [15,16]. Reversely, several studies have not demonstrated an increased rate of hypoglycemia during intensive insulin therapy [17-19]. In this observation, the metabolic improvement was not associated with a higher risk of severe hypoglycemia. Before the course, 18.9% of the patients suffered at least of two episodes of severe hypoglycemia in the year preceding the course and 83.8% patients experienced a mild hypoglycemia. After the course, no episode of severe hypoglycemia was reported and mild hypoglycemia decreased significantly from 11.7 episodes/3 months to 1.7 episodes/3 months at 9 months. This important improvement could be correlated to the well control of self-management skills taught to patients in order to adapt their fast acting insulin doses to pre meal glucose value, meals and physical activity. Our results were consistent with another study, which found that the improvement of the baseline HbA1c was associated to a reduced rate of severe hypoglycemia [20].

The weight gain was described as the second negative effect of intensive insulin therapy besides hypoglycemia [21]. In our study population, there was a slight increase in BMI despite the large nutritional counseling received before the course. The flexible insulin therapy is considered as a crucial turning point in the management of type 1 diabetes. Many teaching programmes included a 5 days training of inpatient or outpatient were published [22,23]. An inpatient five consecutive days programme was adopted in our department and adapted to our patient´s profile. These 5 days teaching course allowed type 1 diabetic patients to focus on diabetes and enlarge their capacity of self-management to lead to a stronger glycemic control and a better quality of life. The educational programme included a nutritional advice adapted to our Moroccan culinary culture provided by a nutritional session during the course and an illustrated detailed booklet designed by our team. During the follow up period of 9 months, our patients have decreased unsustainably their HbA1c. However, there was a greater reduction of hypoglycemic events.

## Conclusion

This report can demonstrate that flexible insulin therapy can be adopted in countries with weak or intermediate income (e.g. Morocco). Beyond the glycemic control, this therapeutic approach has indisputably several advantages. Patients educated to flexible insulin therapy improves their glycemic control without increasing hypoglycemic events.

### What is known about this topic

- Good glycemic control is the main purpose in the management of type 1 diabetes, which prevents patients from macro and microvascular diseases;
- The flexible insulin therapy is an educational programme, which offers to patients with type 1 diabetes the possibility to determine correctly the dose of the fast-acting insulin in order to prevent hypoglycemia and hyperglycemia;
- Many inpatient and outpatient training programmes for flexible insulin therapy were developed in various countries to improve the metabolic control of patients with type 1 diabetes.

### What this study adds

- This study was successful to demonstrate that an adapted educational programme for flexible insulin therapy can improve the metabolic control of patients with type 1 diabetes;
- The specific educational programme of FIT developed in our department was adapted to Moroccan culture and our varied culinary traditions;
- The metabolic control was obtained in our study's population without increasing the risk of hypoglycemia.
